# Outcome prediction for critical care patients with respiratory neoplasms using a multilayer perceptron neural network

**DOI:** 10.31744/einstein_journal/2023AO0071

**Published:** 2023-08-28

**Authors:** Beatriz Nistal-Nuño

**Affiliations:** 1 Complexo Hospitalario Universitario de Pontevedra Pontevedra PO Spain Complexo Hospitalario Universitario de Pontevedra, Pontevedra, PO, Spain.

**Keywords:** Artificial neural network, Intensive care units, Respiratory tract neoplasms, Survival, Hospital mortality

## Abstract

**Objective:**

The variation in mortality rates of intensive care unit oncological patients may imply that clinical characteristics and prognoses are very different between specific subsets of patients with cancer. The specific characteristics of patients with cancer have not been included as risk factors in the established severity-of-illness scoring systems and comorbidity scores, showing limitations in predicting mortality risk. This study aimed to devise a predictive tool for in-hospital mortality for adult patients with a respiratory neoplasm admitted to the intensive care unit, using an artificial neural network.

**Methods:**

A total of 1,221 stays in the intensive care unit from the Beth Israel Deaconess Medical Center were studied. The primary endpoint was the all-cause in-hospital mortality prediction. An artificial neural network was developed and compared with six severity-of-illness scores and one comorbidity score. Model building was based on important predictors of lung cancer mortality, such as several laboratory parameters, demographic parameters, organ-supporting treatments, and other clinical information. Discrimination and calibration were assessed.

**Results:**

The AUROC for the multilayer perceptron was 0.885, while it was <0.74 for the conventional systems. The AUPRC for the multilayer perceptron was 0.731, whereas it was ≤0.482 for the conventional systems. The superiority of multilayer perceptron was statistically significant for all pairwise AUROC and AUPRC comparisons. The Brier Score was better for the multilayer perceptron (0.109) than for OASIS (0.148), SAPS III (0.163), and SAPS II (0.154).

**Conclusion:**

Discrimination was excellent for multilayer perceptron, which may be a valuable tool for assessing critically ill patients with lung cancer.

## In Brief

Nistal-Nuño devised a predictive tool for in-hospital mortality for adult patients with a respiratory neoplasm admitted to the intensive care unit using an artificial neural network. Specific features of patients with cancer and sequential laboratory parameters at four time points were included as risk factors in the model, unlike the traditional severity-of-illness scoring systems and comorbidity scores.

## Highlights

Two artificial neural networks, type of multilayer perceptron, and a random forest model were developed
Multilayer perceptron and random forest were compared to the OASIS, SAPS, SAPS II, SAPS III, LODS, SOFA, and EVCI Scores
Discrimination was assessed with the receiver operator characteristic and precision-recall curves.
Calibration of the predictive models was evaluated with the Brier Score

## Graphical abstract

**Figure:**
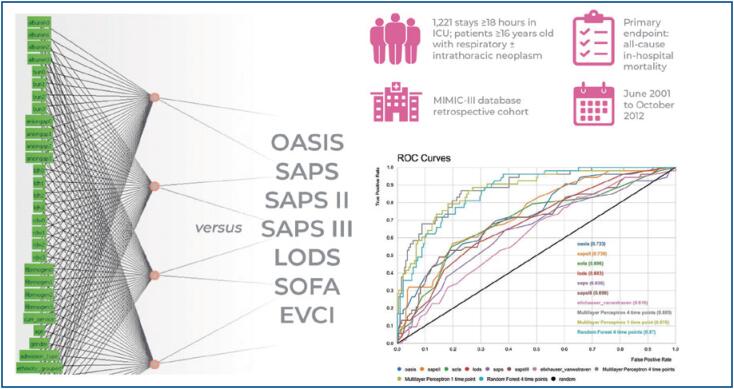

**Outcome prediction for critical care patients with respiratory neoplasms using a multilayer perceptron neural network**

## INTRODUCTION

Neoplasms of the respiratory tract are one of the most frequently diagnosed cancers and the leading cause of cancer-related deaths worldwide.^(1)^ An increasing number of patients with lung cancer are at risk of admission to an intensive care unit (ICU) due to cancer-related complications or treatment complications.^(2,3)^ Considering the high incidence of respiratory neoplasms and their negative prognosis, it would be highly beneficial to develop effective clinical predictors of short-term mortality for ICU patients with lung cancer^(4)^ in order to help clinicians to identify lung cancer patients at high risk of mortality influencing clinical decisions to improve outcomes.

Scoring systems that measure the severity of illness have been developed for the general population of ICU patients. These traditional systems are widely established and are used to assess the gravity of critical illness and predict mortality. These include the Logistic Organ Dysfunction Score (LODS),^(5)^ Oxford Acute Severity-of-Illness Score (OASIS),^(6)^ Simplified Acute Physiology Score (SAPS),^(7)^ SAPS II,^(8)^ SAPS III,^(9)^ and Sequential Organ Failure Assessment (SOFA).^(10)^

Comorbidity scores have also been generated for the general population of ICU patients, such as the Elixhauser-van Walraven Comorbidity Index (EVCI).^(11)^The EVCI is based on 30 acute and chronic comorbidities to predict in-hospital mortality in ICU patients.^(12)^The Elixhauser Score was revised in 2009 by Van Walraven et al. into a weighted scoring system.^(13)^In contrast to the previous systems, EVCI can be computed at the moment of ICU admission and does not require the assessment of laboratory and bedside clinical information.^(11)^

However, these general ICU scores were not specifically developed for patients with cancer. Studies validating the predictive capabilities of traditional ICU scoring systems among ICU patients with cancer suggest that their ability to predict mortality remains suboptimal.^(3)^

Additionally, previous research has found highly varied in-hospital mortality for patients with cancer.^(14)^ This variation in mortality rates may imply that clinical characteristics and prognoses are very different between specific subsets of patients with cancer.^(14)^ Therefore, not only do patients with cancer need specific mortality predictor tools compared to the general ICU patients, but also specific subsets of ICU patients with cancer would benefit from scoring systems targeted to their specific subpopulation, such as the subset of patients with lung cancer.

Several prognostic parameters have been recognized as potential predictors of short-term mortality in patients with lung cancer. One such parameter is blood urea nitrogen (BUN).^(1)^

Cancer-associated hypercoagulable conditions, inflammation, and malnutrition are common in patients with cancer. Moreover, they are closely linked to cancer initiation, progression, and metastasis.^(15)^ The plasma fibrinogen level increases in a hypercoagulable and inflammatory state.^(16)^ Serum albumin has been shown to be a prognostic factor in lung and other cancers.^(17)^ Wen et al. found that fibrinogen-to-albumin ratio was an independent prognostic factor for all-cause cancer mortality.^(18)^ Therefore, BUN, albumin, and fibrinogen were selected for the developed model.

Several investigations have reported that red blood cell distribution width (RDW) is associated with mortality in ICU patients with cancer^(4)^ and patients with lung cancer.^(19,20)^ Lactate dehydrogenase (LDH) is considered as a relevant prognostic biomarker in neoplastic diseases,^(21,22)^ including lung cancer.^(23)^Therefore, RDW and LDH were selected for the developed model.

The laboratory parameters described have important prognostic significance for patients with cancer; however, they are not included in any traditional ICU-related or comorbidity scores. These scores are broadly utilized in general ICU patients, but may be less accurate in the case of ICU patients with cancer.^(3)^Therefore, the developed model intends to provide better predictive performance than the general ICU scoring systems. Additional features that have been demonstrated to have an important prognostic value for mortality in ICU patients with cancer were included in the developed model.

The developed model uses an artificial intelligence approach to increase predictive performance compared to traditional systems. The traditional systems mentioned previously use logistic regression or a weighted summation of scores, except for OASIS which was created using machine learning algorithms of type particle swarm optimization.^(6)^Logistic regression has several disadvantages. For example, nonlinear problems cannot be solved adequately with logistic regression because logistic regression has a linear decision surface, and linearly separable data are rarely found in medical scenarios. Advanced algorithms such as artificial neural networks (ANN) have overcome their limitations.

An example in the literature on using artificial intelligence for ICU patients with cancer is the study of Santos et al. The study compared the predictive capabilities of artificial intelligence algorithms to estimate the risk of quality-adjusted life years of ≤30 days for 777 patients in ICUs of two Brazilian public hospitals specialized in cancer care. Except for the decision trees, the predictive models derived from machine learning were almost equivalent, presenting good discrimination.^(24)^

To date, no artificial intelligence method has been developed to predict short-term mortality for ICU patients with lung cancer. Artificial neural networks are especially appropriate for multivariate datasets with nonlinear dependencies and they do not need variables to fit any theoretical distribution. In contrast to the static traditional severity-of-illness systems, the developed ANN captures the dynamic variation in laboratory parameters over time in the ICU. The short-term prognosis of in-hospital mortality reflects the realistic goals of clinicians treating patients in the ICU.

## OBJECTIVE

This study aimed to devise a predictive tool for all-cause in-hospital mortality for individual adult patients with a respiratory neoplasm admitted to the intensive care unit, using an artificial neural network.

## METHODS

### Data source and study population

Data were obtained retrospectively from the Medical Information Mart for Intensive Care (MIMIC)-III critical care database version v1.4. per the ethical guidelines of the Institutional Review Board of the Beth Israel Deaconess Medical Center (BIDMC) and the Massachusetts Institute of Technology. The MIMIC-III database is a large dataset containing de-identified clinical data of individual patients admitted to ICUs between June 2001 and October 2012 at the BIDMC (United States).^(25)^

The study included all ICU patients admitted with at least one diagnosis of a respiratory and/or intrathoracic neoplasm according to the corresponding International Classification of Diseases (ICD)-9 codes,^(2)^ under any hospital service. Since all patient diagnoses were sequenced by priority in the MIMIC dataset, having a diagnosis code of a respiratory neoplasm could be at any diagnosis position.

In addition, adult patients aged ≥16 years with a length of ICU stay and survival ≥18 hours following ICU admission and all admissions to the ICU for a patient were included in the study. A total of 1,221 ICU stays were recorded for patients who met the previous criteria and were used as the final cohort. The threshold of 18-hours length of stay was selected to permit the extraction of laboratory parameters at four time points during the ICU stay. Code in PostgreSQL language generated for selecting the ICU stays is available at.^(26)^

The primary endpoint was all-cause in-hospital mortality prediction, for the same hospital admissions of the corresponding ICU stays. For this primary outcome, the ANN was compared with the OASIS, SAPS, SAPS II, SAPS III, LODS, SOFA, and EVCI Scores. Developed code from the MIMIC Code Repository was used to generate the previous scores for the studied population.^(27)^

### Variables extracted and processing

The extracted variables were laboratory parameters measured at four consecutive time points and categorical patient features (Table 1). The laboratory variables were serum albumin, BUN, serum anion gap, blood LDH, RDW, and fibrinogen levels. The four time points when these values were extracted were at ICU admission and at the 6-, 12-, and 18-hours after ICU admission. In a secondary analysis, for a fair comparison between the ANN and traditional systems, only available features at the time of ICU admission were considered (one time point).

**Table 1:** Patient variables obtained for constructing the machine learning models with four time points. Categorical features are attributes, except for patient age which is a continuous measure. The traditional systems compared use a few of these features as well

Sequential features obtained at four time points (continuous features)*			Categorical features*
Laboratory#	Albumin (g/dL), BUN (mg/dL), anion gap (mEq/L), LDH (IU/L), RDW (%), Fibrinogen (mg/dL)	Type of ICU admission (admission_type)	Elective, urgent, or emergency
		Ethnicity	White, Black, Hispanic, Asian, Native, Unknown, other
		Sex	
		Age at ICU admission	
		First hospital clinical service that the patient was admitted under (service)	Cardiac Medical; Cardiac Surgery; Dental; Ear, nose, and throat; Genitourinary; Gynecological; Medical; Neurologic Medical; Neurologic Surgical; Obstetrics; Orthopaedic; Orthopaedic medicine; Plastic; Psychiatric; Surgical; Trauma; Thoracic Surgical; Vascular Surgical
		Sepsis	Patients with a primary diagnosis of sepsis were identified using the Angus methodology,^(29)^ which is based on ICD-9 codes for either a bacterial or fungal infection in combination with acute organ dysfunction
		Vaso_flag	Norepinephrine, epinephrine, phenylephrine, vasopressin, dopamine, isoprenaline
		RRT	Renal replacement therapy
		Vent	If patients received any mechanical ventilation “events” during their ICU stay.
			Certain elements end the current ventilation event:
			a) documented extubation ends the current ventilation
			b) initiation of non-invasive ventilation and/or oxygen ends the current ventilation
		Summary_dnr	CMO= comfort measures only
			DNR= do not resuscitate
			DNI= do not intubate
			DNCPR= cardiopulmonary resuscitation not indicate
		Metastatic	Metastatic cancers were identified independently of the anatomic site based on ICD-9 codes^(2)^

* All features are normalized before being used as input by the machine learning models becoming continuous, including the nominal attributes;

# The interval between each time point was a 6-hour window, except for the first time point that allowed collection of laboratory parameters from 24 hours backwards rendering them available at the time of ICU admission.

BUN: blood urea nitrogen; LDH: lactate dehydrogenase; RDW: red blood cell distribution width; ICU: intensive care unit.

Serum albumin was included, as it has been shown to be a prognostic predictor of mortality in lung cancer^(1,17)^ and general patients with cancer.^(18)^ Blood urea nitrogen was selected for the same reasons.^(1)^ The serum anion gap was selected because it is a general predictor of mortality in the ICU. Lactate dehydrogenase was selected as it has been demonstrated to be a negative prognostic marker in lung cancer^(23)^ and several tumors.^(21,22)^Red blood cell distribution width was selected as it has been shown to be a prognostic factor of short-term mortality following hospitalization in lung cancer.^(4,19,20)^ Fibrinogen was included, as it has been proposed that it may predict the probability of cancer mortality.^(15,16,18)^ Typical serum tumor markers used in lung cancer prognosis, such as carcino embryonic antigen and cancer antigen 125, were not included because these are not usually measured in the ICU.^(23)^

The categorical features obtained included demographic parameters, organ-supporting treatments, and clinical information. Among the demographics, age at ICU admission was included as it is a traditional prognostic marker for mortality.^(1,4,14,23)^ Sex was also included as a traditional prognostic marker.^(23)^ Ethnicity was included, as it is an important patient characteristic associated with outcomes.^(2,4)^ The admission type was also included, as it has been shown to be an important characteristic affecting mortality.^(2,14)^

The obtained clinical information features included the first hospital service under which the patient was admitted. Evidence shows that the clinical service provider for ICU patients with cancer impacts mortality.^(2,14)^ The variable of do-not-resuscitate order was selected as supported by Sauer et al.,^(2)^ including several code statuses described in table 1, given at any time through the ICU stay. The metastatic variable was included, which was reported to be associated with mortality.^(1,14,28)^ The variable sepsis was included as it has been demonstrated to negatively affect cancer mortality in ICU.^(1-3,29)^

Among the organ-supporting treatments, vasopressor use was included, indicating whether a patient was on a vasopressor during their ICU stay. This is frequently regarded as affecting mortality.^(1-3,14)^ The utilization of renal replacement therapy at any time during ICU stay was included, as reported as a clinical factor associated with mortality in patients with cancer.^(1-4,14,28)^ The use of mechanical ventilation at any time during ICU stay was included as an important prognostic variable for ICU patients with cancer.^(1-3,14)^

**Figure 1:**
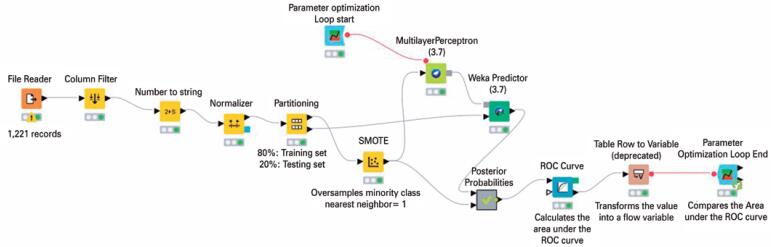
KNIME workflow design used to build the multilayer perceptron. The values of the measurements obtained and categorical patient features were used to represent the values of the input neurons of the multilayer perceptron after they were normalized. The value representing the primary outcome was used to describe the activity of the output neuron

Konstanz Information Miner *(KNIME AG, Zurich, Switzerland)*^(30)^ was used to build the machine learning models. The input dataset was split by stratified sampling into two partitions: 80% for training and 20% for testing (Figure 1). The machine learning models were built with the training data and their performance was evaluated on the testing set. The training set was resampled using a Synthetic Minority Oversampling Technique to balance the target class, and the predicted class probabilities were corrected based on the *a priori* class distribution of the data. The same testing set (n=245) was used to assess the performance of all models.

### Multilayer perceptron model

The ANN used was a multilayer perceptron (MLP) based on WEKA 3.7, which uses backpropagation to classify the instances. The MLP is a feedforward-network without shortcut connections. The backpropagation algorithm has the learning parameters specified in table 2, which were optimized through a loop (Figure 1) that attempts to maximize the area under the receiver operator characteristic curve (AUROC) during the simulations for the primary outcome. The best parameter values obtained during the simulations are listed in table 2. The MLP models were compared in performance to other machine learning model, a random forest (RF), which also used four time points as the main MLP model.

**Table 2:** The best parameters found during the optimization loops for in-hospital mortality prediction for the multilayer perceptron model developed with four time points

	Multilayer perceptron (four time points)
Learning rate parameter *η*, which indicates the step width of the gradient descent^(30)^	0.41
momentum term *μ* applied to the weights during updating^(30)^	0.91
Training time: the number of epochs to train through^(30)^	2,881
Validation set size: the percentage size of the validation set to use to terminate training^(30)^	51
Validation threshold: the consecutive number of errors allowed for validation testing^(30)^	26
Hidden neurons of the hidden layer of the network	4
Learning rate decay will occur^(30)^	True

### Performance measures

Discrimination was assessed using receiver operating characteristic (ROC) curves, AUROC, precision-recall curves (PRC), and area under the precision-recall curve (AUPRC). Precision-recall curves provide a measure of performance that ignores the number of true negatives and can be useful for problems with class imbalance, as in this population.

The null hypothesis was set *a priori* as that there are no differences in discriminatory capability among the machine learning models and the severity-of-illness systems and comorbidity score compared. Pairwise comparisons of all ROCs and PRCs were used to test the statistical significance of the discriminatory differences between the machine learning models and traditional systems. The difference between the AUROCs was calculated using the DeLong method. The level of significance was set at a two-sided p<0.05.

Hypothesis testing and calculation of AUPRC were performed using MedCalc^®^ Statistical Software version 20.027 (MedCalc Software Ltd, Ostend, Belgium; https://www.medcalc.org; 2022).

The Brier Score was used to assess the calibration of the predictive models. This was computed for the machine learning models, OASIS, SAPS II, and SAPS III.

## RESULTS

Of the 1,221 ICU stays, 262 resulted in death during the same hospital admission of the corresponding ICU stay, and 959 resulted in survival, representing a prevalence of 21.457% for in-hospital mortality.

**Figure 2:**
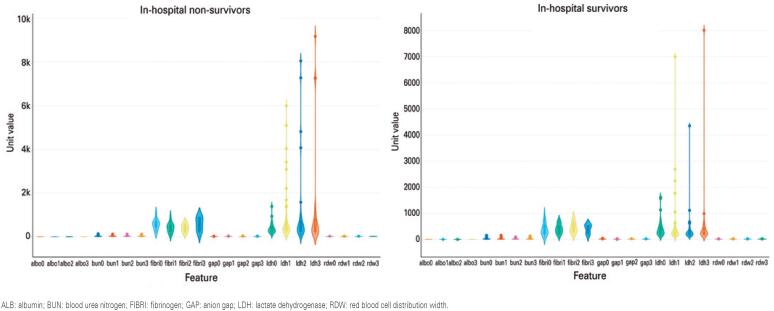
Violin plots showing the comparisons of laboratory parameters between the cohort of survivors (right) and non-survivors (left) in-hospital. Each violin plot displays a traditional boxplot with quartile notations for each feature, mean, median, as well as single points for outliers. Features’ numerical values are represented on the y-axis, which correspond to the unit of measurement for each parameter as detailed in table 1

The violin plots in figure 2 show comparisons of the laboratory parameters analyzed between the cohort of survivors and non-survivors in-hospital. A greater variation in laboratory parameters was observed for fibrinogen and LDH in survivors and non-survivors, where violin shapes were more clearly displayed. Regarding these particular violins, we can observe that the values for the non-survivors are higher than for the survivors and also seem to increase over time for the non-survivors.

**Figure 3:**
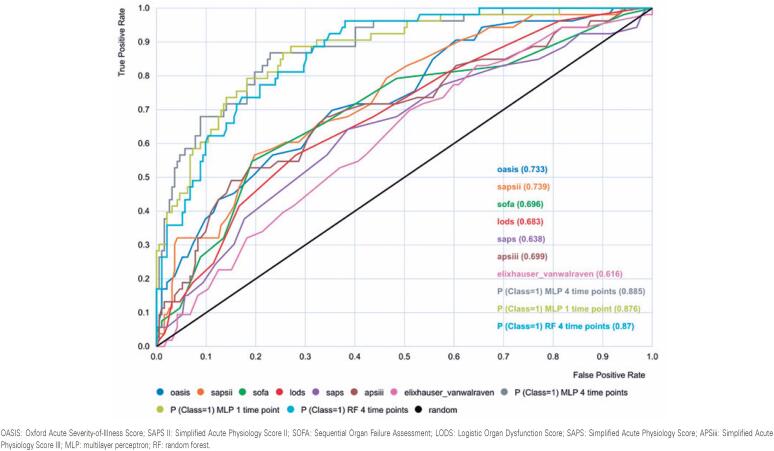
Receiver operator characteristic curves for in-hospital mortality prediction for the machine learning models built, severity-of-illness systems, and Elixhauser-van Walraven Comorbidity Index compared

Figure 3 displays the ROC curves for the machine learning models, severity-of-illness systems, and EVCI, which show an AUROC of 0.885 for MLP (four-time points), 0.876 for MLP (one-time point), 0.87 for RF, and ≤0.739 for the conventional systems (Table 3).

**Table 3:** Comparison of performance between the machine learning models, severity-of-illness systems, and Elixhauser-van Walraven Comorbidity Index

	MLP(four-time points)	MLP(one-time point)	RF(four-time points)	OASIS**	SAPS III**	SAPS II**	LODS**	SOFA**	EVCI	SAPS**
AUROC* for in-hospital mortality (95%CI)#	0.885	0.876	0.87	0.733	0.699	0.739	0.683	0.696	0.616	0.638
	(0.836-0.934)	(0.824-0.928)	(0.821-0.918)	(0.657-0.809)	(0.615-0.784)	(0.666-0.813)	(0.604-0.762)	(0.612-0.779)	(0.533-0.699)	(0.551-0.725)
AUPRC* for in-hospital mortality (95%CI)†	0.731	0.717	0.67	0.482	0.429	0.452	0.355	0.406	0.277	0.341
	(0.596-0.833)	(0.582-0.821)	(0.534- .782)	(0.352-0.614)	(0.304-0.565)	(0.324-0.586)	(0.239-0.492)	(0.283-0.542)	(0.173-0.411)	(0.227-0.477)
Brier Score‡ for in-hospital mortality*	0.109	0.116	0.139	0.148	0.163	0.154				

* Results shown were calculated from test data (n=245);

# The 95%CI was calculated as AUROC±1.96 standard error;

† The 95%CI was calculated with the Logit method;

‡ The Brier Score was calculated as the mean squared error of the prediction;

** The severity-of-illness systems’ scores are calculated from the first 24 hours of intensive care unit stay, except for the SAPS III which requires 1 hour.

MLP: multilayer perceptron; RF: random forest; OASIS: Oxford Acute Severity-of-Illness Score; SAPS III: Simplified Acute Physiology Score III; SAPS II: Simplified Acute Physiology Score II; LODS: Logistic Organ Dysfunction Score; SOFA: Sequential Organ Failure Assessment; EVCI: Elixhauser-van Walraven Comorbidity Index; SAPS: Simplified Acute Physiology Score; AUROC: area under the receiver operator characteristic curve; 95%CI: 95% confidence interval; AUPRC: area under the precision-recall curve.

**Figure 4:**
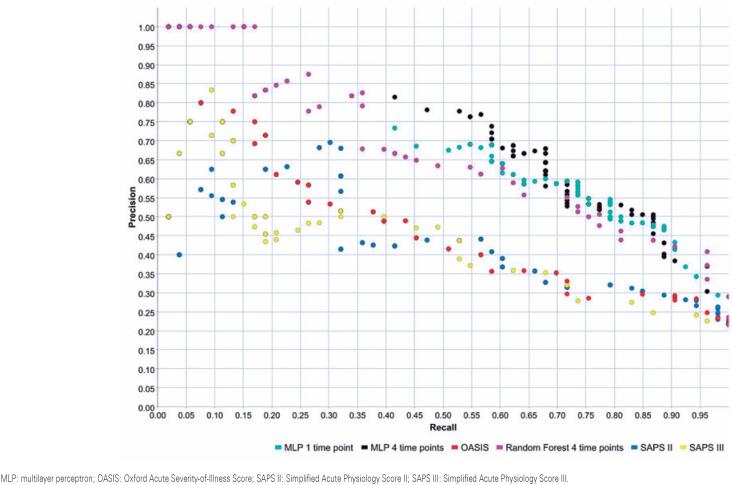
Precision-recall curves for the machine learning models built, SAPS II, OASIS, and SAPS III for in-hospital mortality prediction

Figure 4 shows the PRCs for the machine learning models, SAPS II, OASIS, and SAPS III, which yielded an AUPRC of 0.731 for MLP (four-time points), 0.717 for MLP (one-time point), 0.67 for RF, and ≤0.482 for the traditional systems (Table 3).

The pairwise comparisons of all AUROCs between the machine learning models and the traditional systems are presented in table 4. The machine learning models were substantially superior to all conventional systems, with p≤0.0001 for all comparisons (Table 4).

**Table 4:** Pairwise comparisons of all AUROC and AUPRC between the machine learning models and the severity-of-illness systems and Elixhauser-van Walraven Comorbidity Index for predicting in-hospital mortality

	AUROC* †			AUPRC* #		
	MLP (four-time points)	MLP (one-time point)	RF (four-time points)	MLP (four-time points)	MLP (one-time point)	RF (four-time points)
OASIS	95%CI=0.0831-0.221	95%CI=0.0707-0.215	95%CI=0.0685-0.205	Bootstrap 95%CI =0.1975-0.3010	Bootstrap 95%CI=0.1841-0.2898	Bootstrap 95%CI=0.1506-0.2297
	p<0.0001	p=0.0001	p=0.0001			
SAPS III	95%CI=0.106-0.266	95%CI=0.097-0.256	95%CI=0.0933-0.247	Bootstrap 95%CI=0.2442-0.3505	Bootstrap 95%CI=0.2294-0.3432	Bootstrap 95%CI=0.1935-0.2856
	p<0.0001	p<0.0001	p<0.0001			
SAPS II	95%CI=0.0843-0.207	95%CI=0.0724-0.201	95%=0.0631-0.198	Bootstrap 95%CI=0.2219-0.3287	Bootstrap 95%CI=0.2053-0.3153	Bootstrap 95%CI=0.171-0.2639
	p<0.0001	p<0.0001	p=0.0001			
LODS	95%CI=0.137-0.267	95%CI=0.123-0.262	95%CI=0.118-0.256	Bootstrap 95%CI=0.3316-0.4310	Bootstrap 95%CI=0.3106-0.4129	Bootstrap 95%CI=0.2752-0.3643
	p<0.0001	p<0.0001	p<0.0001			
SAPS	95%CI=0.169-0.325	95%CI=0.156-0.32	95%CI=0.154-0.31	Bootstrap 95%CI=0.3227-0.4474	Bootstrap 95%CI=0.307-0.4285	Bootstrap 95%CI=0.2688-0.3816
	p<0.0001	p<0.0001	p<0.0001			
SOFA	95%CI=0.115-0.264	95%CI=0.104-0.257	95%CI=0.0942-0.254	Bootstrap 95%CI =0.2648-0.3842	Bootstrap 95%CI=0.249-0.3669	Bootstrap 95%CI=0.2129-0.3158
	p<0.0001	p<0.0001	p<0.0001			
EVCI	95%CI=0.176-0.362	95%CI=0.167-0.352	95%CI=0.161-0.346	Bootstrap 95%CI=0.4024-0.5097	Bootstrap 95%CI=0.391-0.4887	Bootstrap 95%CI=0.3414-0.4441
	p<0.0001	p<0.0001	p<0.0001			

* Results shown were calculated from test data (n=245);

† The 95%CI for the difference between two AUROCs was calculated as AUROC difference±1.96 standard error;

# The bootstrap technique was used to calculate the 95%CI of the difference between the AUPRCs.

MLP: multilayer perceptron; RF: random forest; 95%CI: 95% confidence interval; AUROC: area under the receiver operator characteristic curve; AUPRC: area under the precision-recall curve; OASIS: Oxford Acute Severity-of-Illness Score; SAPS III: Simplified Acute Physiology Score III; SAPS II: Simplified Acute Physiology Score II; LODS: Logistic Organ Dysfunction Score; SAPS: Simplified Acute Physiology Score; SOFA: Sequential Organ Failure Assessment; EVCI: Elixhauser-van Walraven Comorbidity Index.

Pairwise comparisons of all AUPRCs between the machine learning models and the traditional systems are presented in table 4. The machine learning models were substantially superior to all traditional systems as the 95% bootstrap confidence intervals did not include 0 (Table 4).

Lower Brier Scores indicate better calibration; it was 0.109 for MLP (four-time points), 0.116 for MLP (one-time point), 0.139 for RF, and ≥0.148 for the traditional systems analyzed (Table 3).

The relative importance of the features in the MLP (four-time points) is presented in table 5.

**Table 5:** The 10 most important features in the multilayer perceptron (four-time points) are displayed in decreasing order from top to bottom

AUROC dropped from 0.885 to	Feature excluded for computation of the AUROC*
0.780464	BUN3
0.801887	Summary_dnr
0.802575	Admission_type
0.809257	BUN2
0.814269	LDH1
0.81987	Aniongap0
0.820067	RDW1
0.82616	Aniongap3
0.827535	LDH3
0.828027	Vent

* The contribution to the overall AUROC for each single feature was evaluated by computing the AUROC when only that feature was excluded.

AUROC: area under the receiver operator characteristic curve; BUN: blood urea nitrogen; LDH: lactate dehydrogenase; RDW: red blood cell distribution width.

## DISCUSSION

Studies carried out on the MIMIC-III database suggest that the survival of overall oncologic ICU patients increased between 2002 and 2011.^(2)^ Although they observed that mortality rates decreased significantly over that period for all patients, there was substantial variation in survival rates among cancer types with hematologic malignancies exhibiting drastic decreases in adjusted mortality rates. However, for solid cancers, the overall improved survival was mainly driven by a drop in genitourinary cancers, while no improvement in respiratory cancers was observed.^(2)^

This is in agreement with the study of Peng et al.,^(1)^ which observed an in-hospital mortality rate of 26.0% in ICU patients with lung cancer in a posterior cohort of patients also at the BIDMC and 26.4% in a posterior cohort at different hospitals. In the current study, the in-hospital mortality prevalence for ICU patients with respiratory cancer was 21.457%. Therefore, until recently, no improvements in survival for respiratory cancers have been observed at the BIDMC. This highlights the need for accurate methods of predicting the mortality risk in patients with respiratory cancer to improve outcomes.

Multilayer perceptron (four-time points) showed the highest AUROC (0.885), followed by MLP (one-time point) and RF. Regarding the AUPRCs, the value for MLP (four-time points) was higher (0.731), followed by MLP (one-time point) and RF. The superiority of machine learning models was statistically significant for all pairwise AUROC and AUPRC comparisons.

The high AUROC and AUPRC for the MLP (four-time points) indicate that its discriminatory capability for predicting in-hospital mortality was excellent, significantly outperforming the conventional systems. Its stronger calibration supports its superiority in this study.

The better performance of the machine learning models is understandable as they capture the specific characteristics of oncological patients admitted to the ICU, especially respiratory cancer. In addition, severity-of-illness systems collect only one time point for the laboratory parameters. Dynamic monitoring of these values may be more accurate. However, when using only one-time point, the performance of the MLP dropped only slightly, indicating that the dynamic monitoring did not have a major impact.

The worst performance was observed for EVCI, which agrees with previous studies that showed low AUROCs for short-term mortality in ICU patients for EVCI.^(11)^ This is mainly because comorbidity scores are not physiology-based like the severity-of-illness systems.

Peng et al. identified the BUN-to-serum albumin ratio as an independent predictor of in-hospital mortality in ICU patients with lung cancer.^(1)^ The relative high significance of BUN was indeed observed in MLP, as it ranked as the 1^st^ and 4^th^ most important feature (Table 5).

Li et al. found that RDW is an independent prognostic factor for short-term mortality in ICU patients with cancer.^(4)^ Red blood cell distribution width is traditionally used to study anemia. Nonetheless, research has demonstrated that RDW is associated with other diseases.^(4)^ Its relative significance was also evidenced in MLP, as *RDW1* ranked 7^th^ in importance (Table 5).

Lactate dehydrogenase is an active enzyme in the anaerobic metabolic pathway. An elevated LDH level has been demonstrated to be a negative prognostic marker for lung cancer.^(23)^ Its relative significance was evidenced in MLP, as LDH1 and LDH3 ranked 5^th^ and 9^th^ respectively in importance (Table 5).

Albumin and fibrinogen are frequently utilized circulating inflammatory proteins.^(16)^ Serum albumin is also a common nutritional parameter. Their relative significance in MLP was lower compared to the top 10 features.

This study had some limitations. Future studies with more detailed lung cancer specific information should be considered to study if the performance of the MLP could be further improved. Traditional prognostic markers such as TNM classification, histopathological features, and patient performance status such as the Eastern Cooperative Oncology Group score could be included. Information about oncological treatment type and time since last administration of chemotherapy could also be included if available.^(2)^

Studies have evidenced that inflammation is linked to tumor progression and metastasis.^(4)^ Among inflammatory indicators, levels of serum C-reactive protein were not analyzed because of the few measurements performed in the population studied. Other parameters closely associated to the inflammatory response which also have been evidenced to play a prognostic role in cancers could be considered such as neutrophil/lymphocyte ratio, platelet/lymphocyte ratio, lymphocyte/monocyte ratio, and interleukin-6.^(4)^ The identification of novel serum biomarkers in lung cancer by proteomics and metabolomics is essential and may help to further refine predictor tools.

This was a single-center retrospective study. Further prospective multicenter studies with larger cohorts are recommended to demonstrate the potential clinical usefulness of the artificial intelligence method proposed.

## CONCLUSION

The performance of the multilayer perceptron developed for prediction of in-hospital mortality for critical care patients with respiratory neoplasms was considerably superior to that of the severity-of-illness systems and comorbidity score compared. The multilayer perceptron provided excellent discrimination and better calibration than the systems compared. The artificial neural network developed might be a good predictor for identifying patients at high risk of in-hospital mortality among critically ill lung cancer patients.
